# Effects of different preparation and cooking processes on the bioactive molecules of *Allium* vegetables

**DOI:** 10.3389/fnut.2024.1350534

**Published:** 2024-06-19

**Authors:** Beyza Katırcıoğlu, Semra Navruz-Varlı

**Affiliations:** ^1^Department of Nutrition and Dietetics, Faculty of Health Sciences, Acıbadem Mehmet Ali Aydınlar University, Istanbul, Türkiye; ^2^Department of Nutrition and Dietetics, Faculty of Health Sciences, Gazi University, Ankara, Türkiye

**Keywords:** onion (*Allium cepa* L.), garlic (*Allium sativum* L.), leek (*Allium porrum* L.), phenolic compounds, preparation, cooking, *Allium* vegetables, bioactive molecules

## Abstract

*Allium* species are among the most widely cultivated vegetables for centuries for their positive effects on human health and their variety of uses in food preparation and cooking. Preparation and cooking processes create chemical changes that can affect the concentration and bioavailability of bioactive molecules. Understanding the changes in bioactive compounds and bioactive activities in *Allium* vegetables resulting from preparation and cooking processes is essential for better retention of these compounds and better utilization of their health benefits. This study aimed to investigate the effects of different preparation and cooking processes on the bioactive molecules of *Allium* vegetables. This review concludes that bioactive compounds in *Allium* vegetables are affected by each preparation and cooking process depending on variables including method, time, temperature. Owing to differences in the matrix and structure of the plant, preparation and cooking processes show different results on bioactive compounds and bioactive activities for different vegetables. Continued research is needed to help fill gaps in current knowledge, such as the optimal preparation and cooking processes for each *Allium* vegetable.

## 1 Introduction

**Figure 1:**
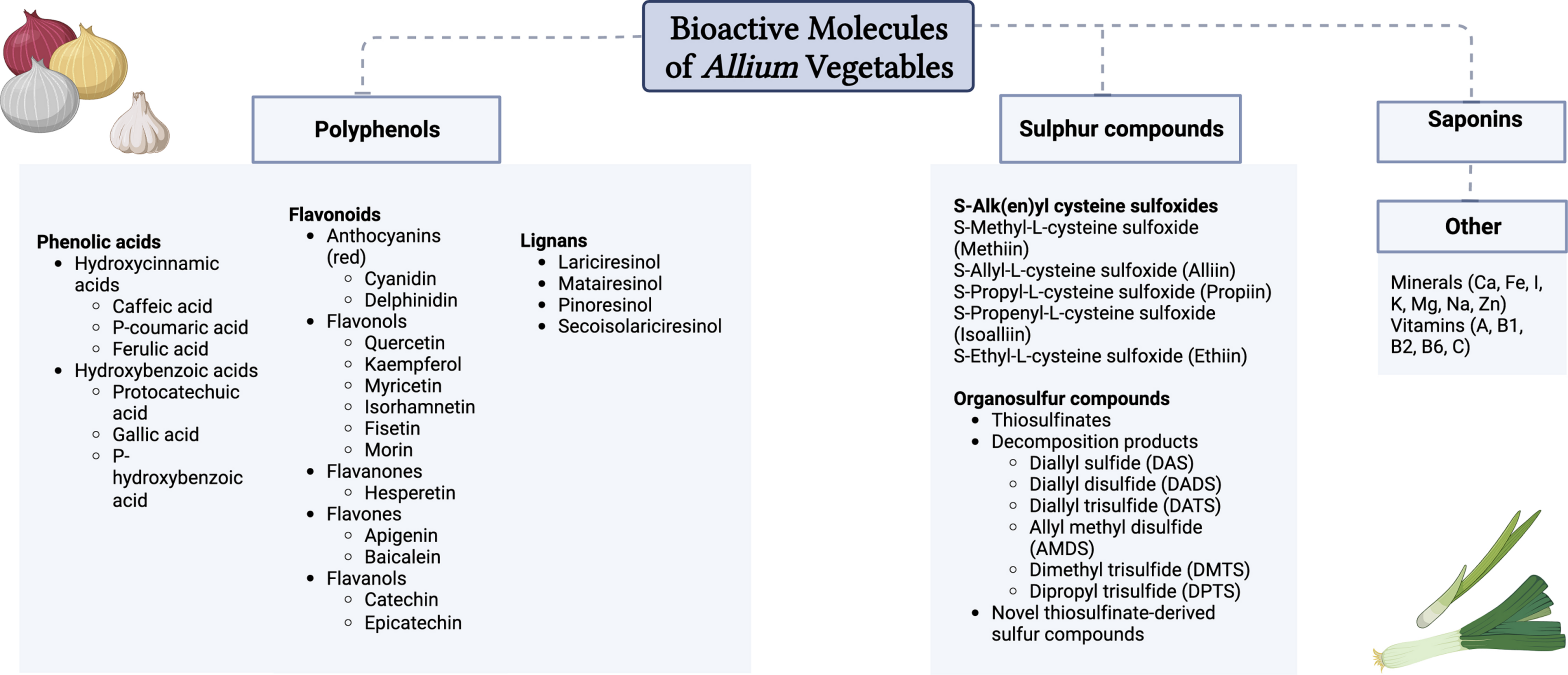
Bioactive molecules in *Allium* vegetables.

*Allium* vegetables belong to the family Alliaceae, including onion (*Allium cepa* L.), garlic (*Allium sativum* L.), leek (*Allium porrum* L.), and Welsh onion (*Allium fistulosum* L.), and contain high levels of bioactive phytomolecules, including phenolic compounds and various organosulfur compounds (1). Owing to their positive effects on human health and their variety of uses in food preparation and cooking, *Allium* species are among the most widely cultivated vegetables for centuries (2). Polyphenols, sulfur compounds, saponins, peptides, vitamins, and minerals (1, 3, 4) are the bioactive molecules contained in *Allium* vegetables (Figure 1). The main bioactive compounds responsible for the health benefits of these vegetables, as well as being the source of their unique taste and odor, are polyphenolic and sulfur compounds (1) (Table 1).

**Table 1:** Nutritional value of *Allium* vegetables (5).

Vegetables (100 g)	Garlic	Yellow onion	Red onion	White onion	Leeks	Welsh onion	Shallots
Energy (kcal)	143.0	38.0	44.0	36.0	61.0	34.0	72.0
Protein (g)	6.6	0.8	0.9	0.9	1.5	1.9	2.5
Total lipid (fat) (g)	0.4	0.05	0.1	0.1	0.3	0.4	0.1
Carbohydrate (g)	28.2	8.6	9.9	7.7	14.2	6.5	16.8
Ca (mg)	181.0	15.0	17.0	21.0	59.0	18.0	37.0
Fe (mg)	1.7	0.3	0.2	0.1	2.1	1.2	1.2
Mg (mg)	25.0	9.0	11.4	9.3	28.0	23.0	21.0
P (mg)	153.0	34.0	41.0	29.0	35.0	49.0	60.0
K (mg)	401.0	182.0	197.0	141.0	180.0	212.0	334.0
Na (mg)	17.0	1.0	1.0	2.0	20.0	17.0	12.0
Vitamin C (mg)	10.0	8.2	8.1	–	12.0	27.0	8.0

**Figure 2:**
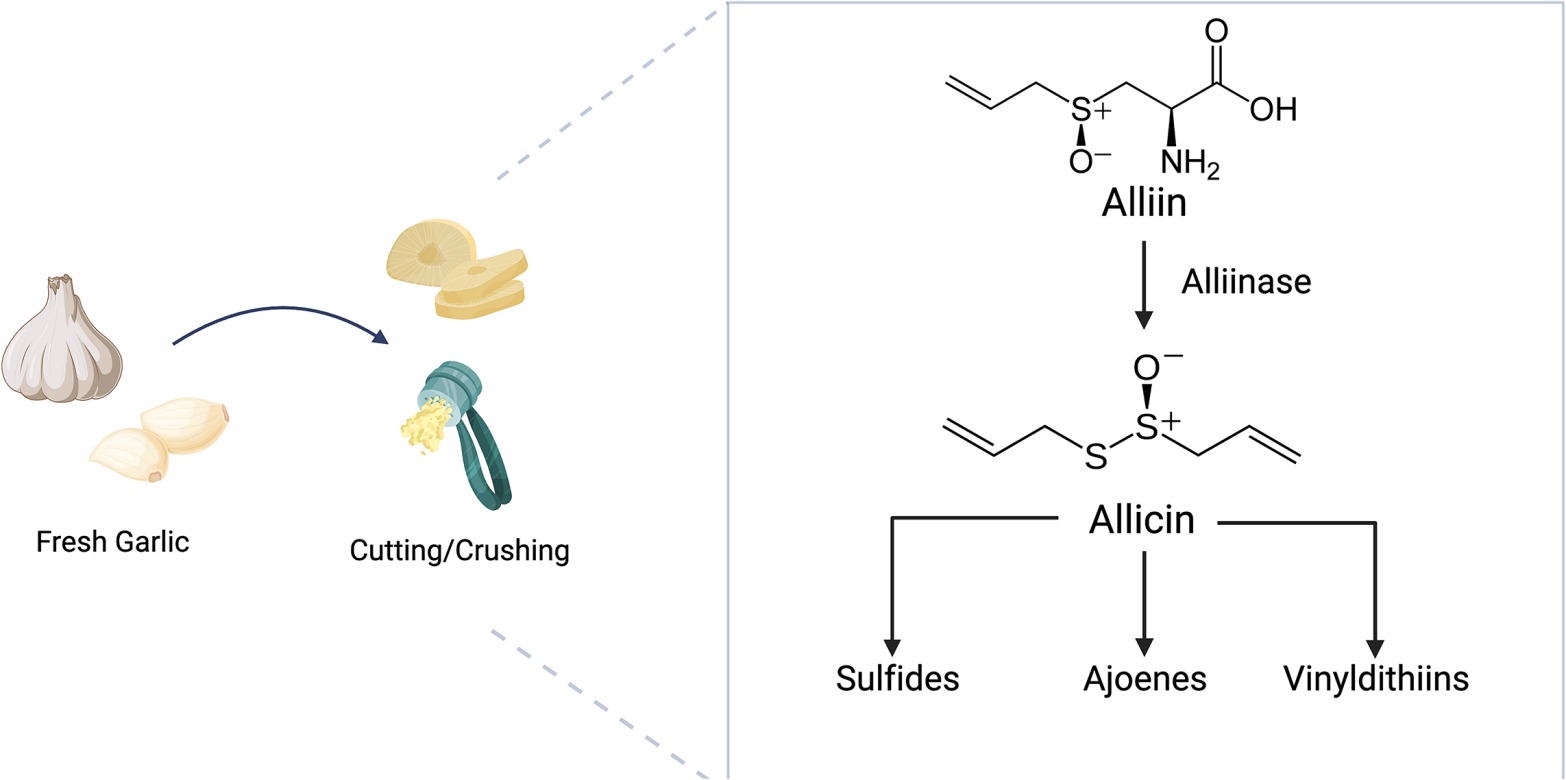
Enzymatic reaction of alliin in the cutting/crushing process of garlic.

Although these plants are normally odorless, up to 50 volatile aroma compounds can be produced during tissue damage through enzymatic hydrolysis of compounds called S-alk(en)yl-L-cysteine sulfoxides (4). Although sulfur compounds account for 85% of the volatile compounds of onion, di and trisulfides are the main compounds of this group (6). Alliin (S-allyl-l-cysteine sulfoxide) is the main component of sulfur compounds in garlic (7). The main bioactive component responsible for garlic’s aroma and functional properties is allicin (diallyl thiosulphinate), which is produced from the reaction of alliin with alliinase when garlic is crushed (8). Diallyl, methyl allyl, and diethyl mono-, di-, tri-, tetra-, penta-, and hexasulfides, vinyldithiins, and sulfur compounds such as (E)- and (Z)-ajoene are produced from unstable allicin (4, 9) (Figure 2). Of these, diallyl trisulfide, diallyl disulfide, and diallyl sulfide exhibit anticancer effects (10).

The active components of *Allium* species particularly garlic and onion have beneficial effects against various diseases, including cardiovascular, neurological, and liver diseases, and are effective in cancer prevention (11). For patients with dyslipidemia, diabetes, and hypertension, garlic is recommended as a long-term dietary component (12). Regular consumption of flavonoid-rich foods helps prevent metabolic syndrome (13). Red onions show varying amounts of inhibitory activity against enzymes involved in the pathogenesis of diabetes, depending on the cooking method and phenolic profile (13). The bioactive compounds quercetin, allicin, and kaempferol, found at high levels in onion, garlic, and leek, respectively, positively affect the immune system (14). Furthermore, leek is effective on cancer cells, and it is suggested that its addition to the diet increases the effectiveness of chemotherapy (15). Furthermore, bioactive proteins and peptides that have shown antioxidative, antimicrobial, anti-cancer potentials and oligosaccharides that have proven potential health benefits as prebiotics are among the bioactive compounds in *Allium* vegetables (16, 17).

Preparation and cooking processes create chemical changes that can affect the concentration and bioavailability of bioactive molecules (18). The matrix and structures of the plant and the characteristics of the compounds are also influential on the changes in bioactive compounds and bioactive activities (19). To identify the appropriate preparation and cooking technique and appropriate measures for preventing losses, recognizing the effects of the process on these bioactive compounds is significant. For example, heat treatment can cause oxidation and other degradation reactions, thereby resulting in the loss of several natural antioxidant contents (20). Furthermore, cooking processes can modulate the release and bioaccessibility of phenolic compounds owing to cell wall softening, cell wall rupture, and release of bound phytonutrients (21, 22). Inappropriate preparation processes result in loss of sensory properties, including taste, appearance, color, consistency, and a decrease in nutritional value and hygienic quality in vegetables (23). To better utilize the nutrient contents of vegetables and fruits, healthy preparation and cooking methods should be applied (23). Understanding the changes in bioactive compounds and bioactive activities in *Allium* vegetables resulting from preparation and cooking processes is essential for better retention of these compounds and better utilization of their health benefits. This study aimed to investigate the effects of different preparation and cooking processes on the bioactive molecules of *Allium* vegetables (Table 2).

**Table 2:** Effects of preparation and cooking methods on the bioactive molecules and antioxidant activity in *Allium* vegetables.

Preparation/cooking method	Type of *Allium* vegetables	Effect	Conclusion	Reference
Washing (chlorine, citric acid, ascorbic acid, nisin, sodium hypochlorite, and calcium chloride)	Onion	↑ TPC ↑ AA	Treating onion with different disinfected has significantly increased TPC and subsequently the strength of antioxidant compounds.	(24)
Washing (citric acid and nisin)	Onion	↓ TPC No differences in quercetin and AA	TPC of onion decreased because of washing with citric acid and nisin; however, phenolic acid and antioxidant activity were not affected by washing	(25)
Water Washing (WW) Hydrogen peroxide (HP) Dichloroisocyanuric sodium salt (DS) Sodium hypochlorite (SH) Sulfuric acid (SA) Citric acid (CA) UV-C radiation	Onion	WW: ↓ Flavonols (%17 at 4°C), (%23 at 50°C); ↓anthocyanins (%29 at 4°C), (%13 at 50°C) HP, DS, SH, SA, CA: Flavonols and anthocyanins did not change significantly compared to the WW UV-C: ↑ flavonol (%35), ↑ anthocyanins (%29)	Flavonols are lost because of solubilization into the immersion water. There is no further loss by oxidation due to the use of decontaminant chemicals. UV-C irradiation is superior to any of the chemical-based procedures	(26)
Cutting	Onion	↓ Propan-1-thiol, (*E*)-1-(prop-1-en-1-yl)-3- propyltrisulfane ↓ 1-(1-(methylthio)propyl)-2-propyldisulfane ↓ (*Z*)- 1-(1-propenyldithio)propyl propyl disulfide ↓ dipropyl trisulfide ↓ methyl 1-(1 propenylthio)propyl disulfide ↑ 2-mercapto-3, 4- dimethyl-2 ↑ 3-dihydrothiophene and 2 ↑ 4-dimethylthiophene	Cutting onions led to a decrease in most of the sulfur compounds	(27)
Cutting	Welsh Onion	↑ TSP ↑ AA	Cutting process of Welsh onion increased the TSP compound and AA	(28)
Crushing	Garlic	↑ dimethyl disulfide ↑ diallyl sulfide ↑ methyl propenyl disulfide ↑ methyl allyl disulfide ↑ diallyl disulfide ↑ diallyl trisulfide No significant differences in Allicin	Most components increased in the headspace between 30 and 240 min after crushing.	(29)
Hot air drying at 50°C–70°C–90°C	Onion	↓dipropyl disulfide ↓dipropyl trisulfide ↓TSC (highest at 90°C) ↑ dimethyl sulfide ↑ dimethyl trisulfide ↑Dimethyl disulfide (only at 90°C) ↑2,5-Dimethylthiophene (only at 90°C)	TSC and dipropyl disulfide decreased during drying, however, most sulfur compounds increased when onion was dried at relatively high temperature.	(30)
Hot air drying	Onion	↓ TPC	The increase in temperature and time resulted in the decrease of TPC	(31)
Hot air drying	White and red onion	↑ TPC ↑ TFC ↑ AA ↑ Gallic acid	TPC, TFC, and AA values of the white and red onions increased significantly.	(32)
Hot air drying at 60°C–70°C	Onion	↑ TSP ↑ AA ↑ Flavonoid	Convective drying increased the TSP, AA and TFC for both temperatures.	(33)
Hot air drying at 40°C (onion rings (OR) and onion flakes (OF))	Onion	TFC: OF> OR VOC: OF> OR	Highlight the different nutraceutical properties of dried onions differing in shape and size.	(52)
Hot air drying	Garlic	↓ TPC ↓ Allicin ↑ Caffeic acid ↑ Ferulic acid ↑ Gallic acid ↑ Quercetin	Individual phenolic compounds was increased while allicin was reduced.	(19)
Sun drying (SD) Hot air drying (HAD) Vacuum drying (VD) Freeze drying (FD)	Onion	↓ TFC ↓ TPC: ↓ Ascorbic acid TFC, TPC and Ascorbic acid retention: FD > VD > HAD>SD	FD: Best drying method for preserving the bioactive composition.	(34)
Vacuum freeze drying (VFD) (Constant temperature at −20°C, 4°C, 25°C, and variable temperature (VT))	Scallion	↓ Allicin ↓ Ascorbic acid ↓ AA Allicin, Ascorbic acid and AA retention: VT > -20 °C> >4 °C > 25 °C	Among the scallion samples with VFD at different main drying temperatures, AA, allicin and ascorbic acid of the VT group was the highest,	(35)
Vacuum freeze drying (VFD) Pulsed vacuum drying (PVD) Hot air drying based on temperature and humidity control (TH-HAD) Air impingement drying (AID) Infrared drying (IRD)	Garlic	Allicin retention: PVD > TH-HAD>AID> VFD > IRD	PVD: have demonstrated best retention on Allicin and SP	(36)
Vacuum freeze drying (VFD) Hot air drying (HAD) Infrared hot air drying (IRHAD) Relative humidity drying (RHD) Pulsed vacuum drying (PVD)	Garlic	↓ Allicin ↓ TPC Allicin and TPC retention: RHD > IRHAD> VFD > PVD > HAD	All drying treatments led to a loss in bioactive compounds of garlic. The IRHAD and RHD dried garlic samples contained significantly higher bioactive compounds than others.	(37)
Freeze drying (FD) Hot air drying (HAD) Vacuum drying (VD) Infrared drying (IRD)	White and purple garlic	↓ Allicin ↓ TPC Allicin and TPC retention: FD > IRD > VD > HAD	FD and IRD treatment can preserve well active substance of white and purple garlic.	(38)
Boiling (BL) Microwaving (MW)	Onion	↑ AA	BL and MW are good ways to retain the antioxidant capacity of onion	(39)
Baking (B) Boiling (BL) Frying (FR) Grilling (GR)	Yellow onion (YO) Red onion (RO)	BL: ↓ TPC, ↓ TFC B, FR, GR: ↑ TPC, ↑ TFC BI for TPC, TFC (YO): B > BL > FR > GR BI for TPC, TFC (RO): B > GR > FR > BL	B and GR: are recommended methods owing to the bioaccessibility of phenolic compounds.	(21)
Deep-frying (DF) Air-frying (AF)	Red onion	DF: ↑ TPC (47.4%) AF: ↑ TPC (18.6%) Bioaccessible TPC after digestion: AF (89.6%) > DF (60.5%) > raw (38.6%)	AF: best for retention TPC after digestion	(40)
Boiling (BL) (5 min-15 min) Steaming (ST)	Leek	ST: ↓ TPC, ↑ AA, ↑ anti-inflammatory activity BL: ↑ TPC, ↑ AA, ↑ anti-inflammatory activity TPC: (BL5 > BL15) anti-inflammatory activity: (BL15 > BL5) AA: (BL15 > BL5)	BL and ST are effective in increasing AA and anti-inflammatory activity	(41)
Cooking at 25°C, 50, 75, 100, 125 and 150°C for 15, 30, 45 and 60 min	Garlic	↑ TPC ↑ TFC	Cooking temperature had a significant effect on TPC and TFC while cooking time did not have a significant effect on the phytochemicals and AA.	(42)
Chopped (Ch) Sliced (Sl) Whole cloves (W) Simmering (S) Rolling boil (RB) Stir- frying (SF)	Garlic	OSCs (all cooking treatments): Ch > Sl > W OSCs (all pre-cooking treatments): SF > RB > S	Best combination treatments to obtain the highest bioactive compound levels were SF-Ch followed by RB-Ch.	(43)
Drying (D) Pickling (P) Frying (F)	Garlic	D, P and F: ↓ TPC, ↓TFC, ↓AA	Drying process caused the highest negative effect on TPC, TFC and AA	(44)
Cooking at 150°C for 20 min	Garlic Onion	↓ AA ↓ PC	Heat treatment had deleterious effects on the antioxidant properties of onion and garlic	(45)
Boiling (B) Frying (F) Roasting (R)	Green onion	↓ TSC	Raw green onions had a higher TSC compared to processed green onions.	(46)

TPC, Total Polyphenol Content; AA, Antioxidant Activity; TSP, Total Soluble Phenolic; TSC, Total Sulfur Compounds; VOC, Volatile Organic Compounds; TFC, Total Flavonoid Content; SP, Soluble Pectin; BI, Bioaccessibility Index; OSCs, Organosulphur Compounds; PC, Phenolic Compounds.

## 2 Search strategy

We searched PubMed, ScienceDirect, and Google Scholar using keywords (“cooking” OR “preparation” OR “washing” OR “drying” OR “cutting”) AND (“bioactive molecules” OR “phenolic compounds” OR “antioxidant activity”) AND (“Allium vegetables” OR “onion” OR “garlic” OR “leek”). Articles written in English were prioritized.

## 3 Preparation and cooking processes of *Allium* vegetables

Vegetables undergo several preparation processes, whether they are to be consumed at home or used in food service systems or industrial products. Domestic processes such as cutting, slicing, peeling, or cooking, as well as thermal and non-thermal industrial processing methods such as high pressure and ultraviolet, can affect some bioactive compounds that are thermally sensitive and vulnerable to chemical or physical processing (47). The sale of fruits and vegetables as fresh cut products has become widespread in recent years owing to the convenience it provides (48). To reduce the biological risk of fresh-cut vegetables and fruits, a chemical wash is performed during fresh-cut produce preparation that serves as a decontamination treatment (49). A sodium hypochlorite aqueous solution (containing approximately 50–200 ppm (parts per million) free chlorine), which is relatively inexpensive and protects against a wide range of microbial agents, is the typical approach for disinfection; however, disadvantages exist in terms of food safety and environmental sustainability (48). Several methods such as ozone and electrolyzed water are used to reduce chlorine exposure and further experiments are conducted with allium vegetables (48, 50). On an industrial scale, peeling is performed mechanically, chemically, or using high-pressure steam peelers, attempting to be as gentle as possible (51).

To extend the shelf life of vegetables, drying is commonly practiced, and the use of dried onions in recipes and as a functional food is becoming widespread (34, 52). The Food and Agriculture Organization (FAO) stated that approximately 106 million tons of dried onions were produced globally in 2021 (53). Hot air drying (HAD) or convective drying, where heat is transferred to the food by hot air in convection ovens, is frequently preferred because it is cost-effective and easy to perform (30). Besides convective drying, other commonly practiced methods include freeze drying (FD), infrared drying (IRD), vacuum drying (VD), and relative humidity drying (RHD). The working principles of these methods are briefly presented. In FD, all moisture in the food is initially frozen and subsequently dehydrated by sublimation; this way, the adverse effects of heat treatment are reduced by using low temperatures (54). When heating is performed by radiation, electromagnetic radiation causes molecules’ thermal movement, thereby causing heat to be transferred by convection and conduction (55). The low pressure created in VD results in a lower boiling point, which helps to avoid the adverse effects of high temperatures (56). In RHD, by reducing the relative humidity of the air during the drying process, the drying time can be shortened, and the loss of phenolic compounds can be reduced (57). Hybrid drying methods, where these drying methods are combined, can be effective in shortening drying time and improving energy efficiency and product quality (58).

## 4 Effects of preparation processes on bioactive molecules

### 4.1 Washing and disinfection process

The nutritional value of fresh-cut fruit and vegetables can be affected by the treatment time, type, and concentration of the disinfection solution (49). Azarpazhooh and Sharayei (24) reported that fresh onion purees prepared with onions washed in different disinfection solutions (chlorine, citric acid, ascorbic acid, nisin, sodium hypochlorite, and calcium chloride) have higher total phenolic compounds and antioxidant activity than the unwashed control group. This may be because of the cutting, which may lead to increased synthesis of sulfur compounds (27).

Chen et al. (25) reported that the total polyphenol content (TPC) of onion decreased because of washing with a solution prepared using citric acid and nisin; however, phenolic acid and antioxidant activity were not affected by washing. The decrease in TPC may be because of the resolution of some onion flavonoids when immersed in water (26). A study conducted by Pérez–Gregorio et al. (26) reported that onion flavonols (both at 4°C and 50°C) passed into the immersion water at a rate of 17–23%. Although disinfection solutions did not lead to further loss of flavonols, anthocyanins were lost at high levels, primarily when hydrogen peroxide was used (26). Conversely, decontamination with ultraviolet (UV)-C irradiation increased natural flavonoid levels in fresh-cut onion slices (26).

The disinfection process can influence bioactive compounds. Dry decontamination processes such as UV-C irradiation are preferable for the preservation of bioactive compounds, as phenolic compounds can pass into the water during washing.

### 4.2 Peeling and cutting processes

The onion’s inner layers have significantly lower phenolic content and antioxidant properties than the outer layers; therefore, removing the outer layers during peeling causes nutrient losses (59). As 90% of quercetin is noted in the onion skin, peeling decreases to 40% in quercetin concentration (60). Although approximately 63% of red onion anthocyanins are observed in the dry peel, the outer fleshy layer, which accounts for 15% of the total weight, is also particularly rich in cyanidin derivatives (61). Only 27% of the total anthocyanins and 79% of quercetin can be consumed when this outer fleshy layer is removed by peeling (25). Onion peel powder-fortified functional foods have received attention for reducing the environmental impact caused by industrial vegetable wastes and benefiting from the bioactive components noted in onion peel (25). A study conducted by Sagar and Pareek (62) showed that TPC, total flavonoid content (TFC), and antioxidant activity increased in fortified bread prepared with 60% whole wheat flour, 40% multigrain flour, and onion peel powder at 1, 2, 3, and 4% concentrations, depending on the enrichment ratio.

The cutting process may result in an increased respiratory rate by ethylene production stimulation (63). Mechanical damage generates a stress signal that may be responsible for various physiological responses, including browning (64). The cutting process can ultimately result in nutritional changes; polyphenol oxidase, which catalyzes polyphenolic compound oxidation, and phenylalanine ammonialase, which catalyzes the synthesis of precursors of phenolic substrates, are responsible for browning (28). A study conducted by Liu et al. (27) revealed that cutting onions led to a decrease in most of the sulfur compounds, whereas 2-mercapto-3,4- dimethyl-2,3-dihydrothiophene and 2,4-dimethylthiophene concentrations increased after cutting. Berno et al. (64) reported that diced onions showed more rapid physiological changes than sliced onions owing to the greater physical stress caused by this type of cutting. A study conducted by Han et al. (28) showed that the cutting process of Welsh onion increased the total soluble phenolic compound and antioxidant activity by increasing wounding intensity.

When garlic is crushed, the unstable allicin rapidly decomposes into various products (9). Varga–Visi et al. (29) reported that sulfur compounds in garlic increased between 30 and 240 min after crushing; however, 10-min boiling hampered the post-treatment formation of sulfur compounds. Therefore, it has been suggested that raw garlic could be crushed and kept in a sealed container during the preparation but should not be left to stand after it is cooked (29).

The bioactive compounds of *Allium* vegetables are influenced by peeling and cutting processes. The bioactive compounds of onions are concentrated in the outer layers and in the onion peel, which is why the removal of these layers during the peeling process leads to bioactive compound degradation. While the positive effect of crushing on the bioactive compounds of garlic is known, wounding can lead to simultaneous production and loss of bioactive compounds in onions.

## 5 Effects of the drying process on bioactive molecules

In a study conducted by Choi et al. (30), dipropyl disulfide, which is the major volatile compound of onion, significantly decreased during drying, whereas most sulfur compounds increased during drying at relatively higher temperatures, such as 90°C. Wongsa et al. (19) reported that the drying temperature and humidity level of the drying air affected bioactive compounds and that the drying process reduced the allicin content and TPC, however, contents of caffeic, ferulic, gallic acids and quercetin increased after drying. This may be because primary phenolic acids are present in a bound form in plant cells and thermal treatment releases these bound phenolic acids (19). Bamba et al. (31) showed that as drying time and temperature increased, TPC decreased owing to thermal degradation. Moreover, compared with unblanched onion powder and fresh samples, blanching of onion bulbs before drying resulted in a loss of approximately 51.4 and 62% of TPC, respectively (31). In a study conducted to evaluate the effect of various pre-treatment methods such as water blanching, ultrasound (US), osmotic treatment (OM), high hydrostatic pressure (HHP) and freeze-thawing (FT) on garlic, it was revealed that allicin content decreased with increasing drying temperature, however, US and OM pre-treatments retained the allicin content better by shortening the drying time (65).

In contrast, Salamatullah et al. (32) reported that drying resulted in an increase in the TPC, TFC, and antioxidant activity of white and red onions. Furthermore, Roman et al. (33) reported that convective drying at 60°C and 70°C increased onion’s total soluble phenolic, antioxidant properties and flavonoid contents. A study conducted by Cecchi et al. (52) showed that onions were cut into two different shapes and subsequently dried at 40°C; onion flakes showed higher flavonoid content and total volatile compounds than onion rings.

A study conducted by Kumari et al. (34) revealed that FD was better than sun drying, HAD, and VD at 55°C ± 5°C; onions dried by FD had intact cell wall structure and were rich in phenolic, flavonoid, and ascorbic acid contents. This may be because of the formation of quinine and H_2_O_2_ by the reaction of O_2_ atoms from the atmosphere with hydrogen atoms from the OH group due to contact with hot air (34). FD onion had the highest ascorbic acid content, whereas a significant decrease in ascorbic acid content was observed in sun drying and HAD owing to termo-sensitive and photo-oxidative reactions (34). A study conducted by Sun et al. (35) showed that the bioactive content ascorbic acid and antioxidant capacity of scallions were better preserved when variable temperatures (from −20°C to −4°C, then to 25°C) were applied during the main drying phase of the VFD process instead of different main drying temperatures (−20°C, −4°C and 25°C).

In a study conducted by Zheng et al. (36), the allicin content of dried garlic was highest in pulsed vacuum drying (PVD) and lowest in IRD. It has been suggested that this can be explained by the fact that drying affects the permeability of plant cell and organelle membranes, thereby causing partial inactivation of alliinase, interrupting allicin synthesis, and leading to allicin destruction (36). Disruption of the cellular structure of garlic increases the likelihood of catalytic hydrolysis, leading to shortening of the cellulose, which was found to be affected by different drying methods, with PVD retaining the SP (Soluble Pectin) best (36). A study conducted by Feng et al. (37) reported that the greatest loss in bioactive compounds of garlic occurred during vacuum-FD and HAD processes, whereas IRD and RHD processes preserved bioactive compounds better. This finding was possibly related to the fact that the drying time of IRD (3.8 h) and RHD (4.6 h) is much shorter than that of vacuum-FD (13.6 h) (at 60°C, excluding vacuum-FD) (37). In a study conducted by Gong et al. (38), FD and IRD processes preserved the active substances of white and purple garlic, including allicin and total phenolics, better than HAD and VD processes (at 60°C except FD). Additionally, dried purple garlic showed significantly higher allicin content, TPC, and antioxidant capacity than dried white garlic (38).

Studies have shown that different drying methods affect bioactive components to different degrees. Drying temperature, drying time, and pre-drying treatments all can influence bioactive compounds. There are conflicting results in the literature regarding the effects of convective drying on the bioactive compound of *Allium* vegetables, this may be because of the difference of the applied temperature in the studies. Although HAD is a practical and frequently used method, hybrid drying methods combining different drying methods and FD, IRD and RHD are better in preserving bioactive molecules by shortening the drying time.

## 6 Effect of cooking process on bioactive molecules

Although cooking is a significant step for food safety, it has a profound impact on the bioactive components and antioxidants of vegetables and can negatively influence the nutritional value if not properly applied (22, 41). During heat treatment, various chemical reactions including oxidative degradation, Maillard reaction, caramelization, and amino acid degradation can occur (46).

One study noted that 30-min boiling, and 15-min microwaving increased the antioxidant capacity of several vegetables, including onions (20). This finding is suggested to be related to the release and activation of antioxidant compounds, including phenolic compounds (20). In a different study, when the effect of *in vitro* digestion on phenolic compounds in yellow and red onions after baking, boiling, frying, and grilling was evaluated, phenolic compounds in baked, fried, and grilled onions increased compared to raw onions, and after *in vitro* digestion, the bioaccessibility of phenolic compounds was highest in baked onions (21). This finding is believed to be because of structural changes that occur during cooking and the release of dietary fiber-bound polyphenols forming the free phenolic compounds (39). In another study, when onion waste-enriched bread was baked, the heat treatment decomposed complex quercetin derivatives, such as dimers or trimers, thereby leading to quercetin release (66). A study conducted by Cattivelli et al. (40) reported that phenolic compounds increased after both air frying (10 min at 200°C) and deep frying (140°C for 10 min in sunflower oil) and that air frying better prevented phenolic compound degradation and showed a high bioaccessibility index in red onion.

A study conducted by İduğ et al. (41) reported that the antioxidant activity and TPC of leek (*Allium ampeloprasum*) increased after cooking, and the antioxidant activity was higher when boiled for 15 min than that when boiled for 5 min. Alide et al. (42) observed that the increase in temperature increased the extraction of bound phenolic compounds and thus increased the antioxidant activity of garlic. This result was valid only if the cooking water was not poured (42). In a study conducted by Locatelli et al. (43), when the effect of different pre-cooking and cooking treatments on the OSCs of garlic was evaluated, chopped raw garlic had the highest allicin concentration which also influenced the levels of the OSCs formed during cooking and stir-frying leads to the generation of more bioactive compounds in garlic than other cooking treatments.

Conflicting results are noted in the literature about the effect of cooking on the bioactive compounds of *Allium* vegetables. A study conducted by Çubukçu et al. (45) reported that cooking in the oven at 150°C for 20 min negatively influenced the phenolic content and antioxidant properties of onion and garlic. This may be because of the oxidation process caused by heat treatment, which may lead to the degradation of the antioxidant components (45). Al-Dabbas et al. (44) also reported a decrease in TPC, TFC and AA concentrations by frying. Another study demonstrated that raw green onions have a higher proportion of sulfur-containing compounds than cooked green onions (46). The cooking process degrades bioactive compounds and oxidizes polyphenols, whereas the Maillard reaction leads to the formation of new compounds, so several interacting reactions during the cooking process seem to lead to conflicting results (28, 67).

Heat treatment affects bioactive compounds in *Allium* vegetables. Cooking process usually increased bioactive compounds. It should be considered that bioactive compounds can pass into the boiling water. Considering the preservation and bioaccessibility of bioactive compounds, pre-cooking chopping, baking, grilling, and frying are among the preferable methods.

## 7 Conclusion

Each of the preparation and cooking processes affects the bioactive components and antioxidant activities of *Allium* vegetables. Owing to differences in the matrix and structure of the plant, preparation and cooking processes show different results on bioactive compounds and bioactive activities for different vegetables. During washing, bioactive compounds can transfer to water. Therefore, dry decontamination processes such as UV-C irradiation are preferred for the preservation of bioactive compounds. The bioactive molecules of onion are more concentrated in the outer layers than those in the inner layers, and the peeling process results in the loss of bioactive compounds that are concentrated in the peel. Removing only the outer dry skin during peeling can help reduce the loss. While the positive effect of crushing on the bioactive compounds of garlic is known, wounding can lead to simultaneous production and loss of bioactive compounds in onions.

Bioactive compounds are influenced by the type of drying process, drying temperature, drying time, and predrying processes. Although HAD is a practical and frequently used method, hybrid drying methods combining different drying methods and FD, IRD and RHD are better in preserving bioactive molecules by shortening the drying time.

The release of phenolic compounds because of heat treatment increases the bioaccessibility of bioactive compounds of Allium vegetables. Of note, bioactive compounds may transfer to the cooking water; these vegetables should be cooked with as little water as possible or in their own water, and cooking water should not be poured. To preserve phenolic compounds and bioavailability, baking, grilling, and frying are recommended. Continued research is needed to help fill gaps in current knowledge, such as the optimal preparation and cooking processes for each Allium vegetable.

## Author contributions

BK: Funding acquisition, Writing – original draft, Writing – review & editing. SN-V: Investigation, Writing – original draft, Writing – review & editing, Funding acquisition, Supervision.
